# Irreducible Dislocation of the Great Toe Interphalangeal Joint Secondary to an Incarcerated Sesamoid

**DOI:** 10.1155/2015/231685

**Published:** 2015-03-22

**Authors:** Hamid Rahmatullah Bin Abd Razak, Zi-Yang Chia, Hwee-Chye Andrew Tan

**Affiliations:** Department of Orthopaedic Surgery, Singapore General Hospital, Outram Road, Singapore 169608

## Abstract

Irreducible dorsal dislocation of the interphalangeal (IP) joint of the great toe is rare. We report a case of a 29-year-old gentleman who presented to the Orthopaedic Surgery Specialist Outpatient Clinic with an irreducible IP joint of the great toe that had been untreated for 4 weeks. The mechanism of injury is believed to be a combination of axial loading with a hyperdorsiflexion force when the patient fell foot first into a drain. As the patient did not report severe symptoms and a true lateral radiograph was not ordered, the dislocation was missed initially at the emergency department. The patient had continued to run and play field hockey prior to visiting us. Incarceration of the sesamoid became a block to manipulation and reduction at the specialist outpatient clinic 3 weeks later. The patient was treated with open surgical exploration, resection of the interposed sesamoid, and Kirschner-wire fixation of the IP joint followed by occupational therapy for mobilization exercises. The operative course was uneventful. At 6 months after surgery, the patient could walk, run, and return to sports.

## 1. Introduction

Dislocation of the interphalangeal joint (IP) of the great toe is an uncommon entity. Most reported cases in the literature are of the metatarsophalangeal joint of the great toe [1]. Irreducible dorsal dislocation of the IP joint of the great toe is rare [1–8]. Closed reduction is often attempted in the emergency setting, but this manoeuvre is seldom successful owing to interposition of the sesamoid bone into the IP joint space. Delayed closed reductions of the IP joint are ineffective because of scarring [9]. Open exploration and reduction is indicated when closed reduction fails [10–14]. In this report, attempts at closed reduction at 3 weeks following initial injury were unsuccessful. The patient underwent open exploration, resection of interposed sesamoid, and Kirschner-wire fixation of the IP joint of the great toe followed by removal of wire at 6 weeks postoperatively and subsequent mobilization. At 6 months following surgery, the patient is able to mobilize his great toe well and has returned to sports. He remains asymptomatic. The patient was informed that data concerning the case would be submitted for publication, and he consented.

## 2. Case Presentation

### 2.1. Clinical History

**Figure 1:**
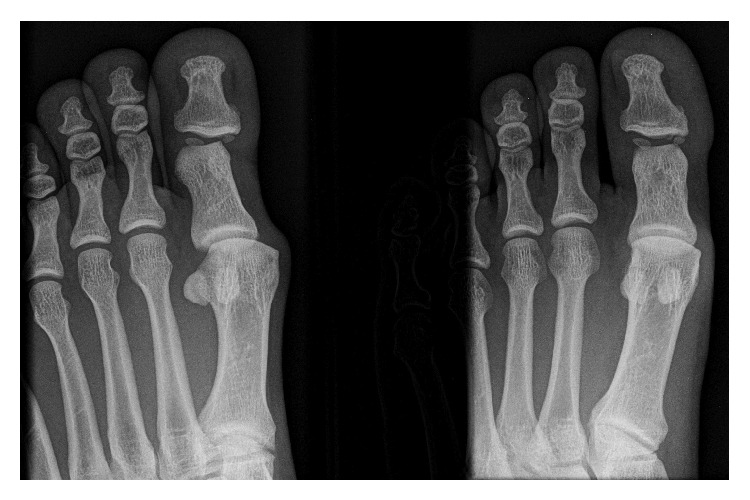
This is the dorsoposterior and oblique radiographs obtained at the emergency department. It reveals an oblique IP joint space of the left great toe, raising suspicion of a dislocation. As a true lateral radiograph was not obtained, the diagnosis was missed.

A 29-year-old gentleman complained of swelling and progressive pain in his left big toe after falling foot first into a drain. From his description of the fall, it seemed like the big toe suffered from an axial loading and hyperdorsiflexion injury. He visited the emergency department of another institution where he was given symptomatic treatment and had dorsoposterior (DP) and oblique radiographs of the left great toe performed (Figure 1). He was told that the radiographs did not reveal any acute fracture and was advised to rest and to attend at the Orthopaedic Surgery Specialist Outpatient Clinic 2 weeks later. The patient felt symptomatically better after a few days and had returned to running and playing field hockey. Three weeks from the initial injury, there was recurrence of pain and swelling in the left great toe and this was limiting the function of the patient. This prompted him to seek attention at our Specialist Outpatient Clinic.

**Figure 2:**
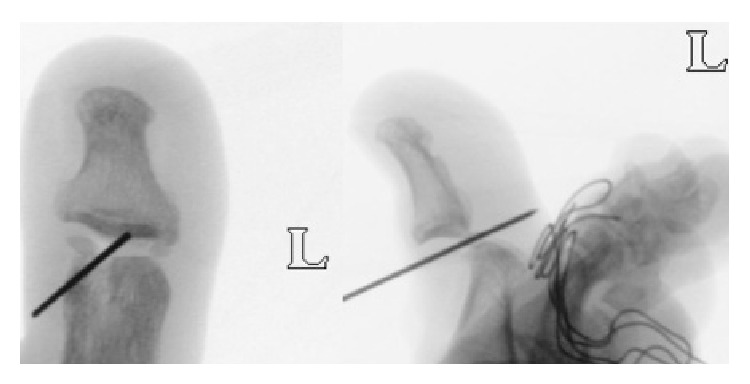
These are intraoperative image-intensifier images of the IP joint of the left great toe showing that interposed sesamoid in the IP joint space caused dorsal dislocation.

On examination, the affected big toe was shortened and dorsally dislocated as compared with the contralateral side. There was tenderness and swelling at the IP joint. While walking, the plantar side of the great toe did not touch the floor. There was lack of active movement of the affected IP joint. The IP joint could not be maneuvered passively too. Normal motion was maintained over the left metatarsophalangeal joint. Plain radiography was repeated and showed obliquity of the IP joint line with an interposed sesamoid bone (Figure 2). Dislocation of the IP joint of the left big toe was suspected based on the clinical presentation as well as subtle features appreciable on the radiographs as discussed with our radiologists. Manual reduction was attempted but was unsuccessful. In spite of advice for surgical reduction, patient was not keen initially as the prescribed analgesics were efficacious in treating the pain and patient was still able to walk and jog without severe symptoms. However, the patient returned 1 week later (4 weeks from initial injury), this time keen for surgical intervention due to persistent swelling and stiffness.

The risks and benefits of surgical intervention were discussed with the patient and informed consent was taken. The patient underwent open exploration, resection of interposed sesamoid, and Kirschner-wire fixation of the left great toe IP joint under general anaesthesia uneventfully as an ambulatory patient.

### 2.2. Surgical Technique

**Figure 3:**
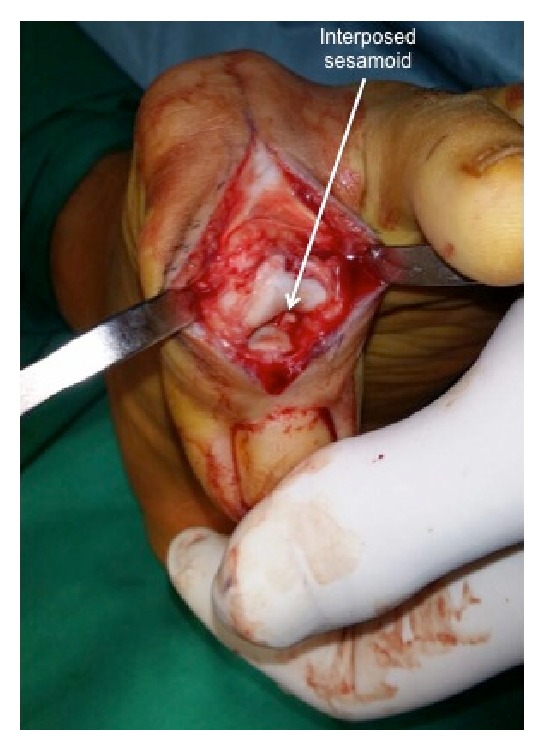
This is a clinical picture depicting open exploration of the IP joint of the left great toe with an interposed sesamoid rendering the dislocation irreducible.

**Figure 4:**
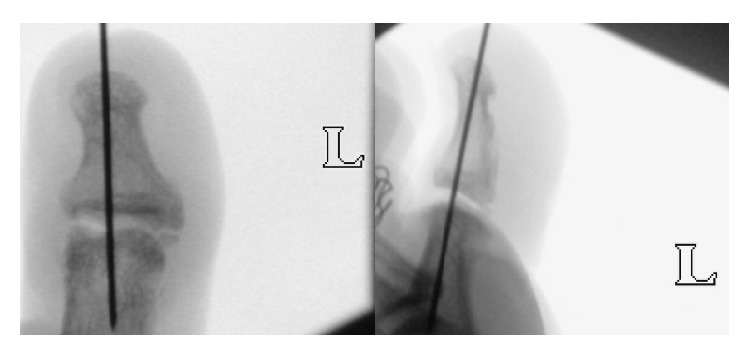
These are intraoperative image-intensifier images of the IP joint being held in reduction by the percutaneously inserted Kirschner-wire.

The left great toe IP joint was approached with a midline dorsal incision and split of the extensor expansion. Intraoperative radiographs confirmed the diagnosis of a dorsal dislocation of the IP joint with an interposed sesamoid (Figure 3). The interposed sesamoid was found in the IP joint space and resected (Figure 4). The IP joint was then ranged to be free from any mechanical block. However, spontaneous reduction of the IP joint was not possible likely due to soft tissue contractures owing to the subacute presentation. Decision was made for temporary fixation of the IP joint by Kirschner-wires. A 2.5 mm Kirschner-wire was driven percutaneously to hold the IP joint in its native reduction. An image-intensifier was used to confirm that the IP joint was in good alignment and reduction. The extensor expansion was repaired and layered closure performed.

### 2.3. Follow-Up

**Figure 5:**
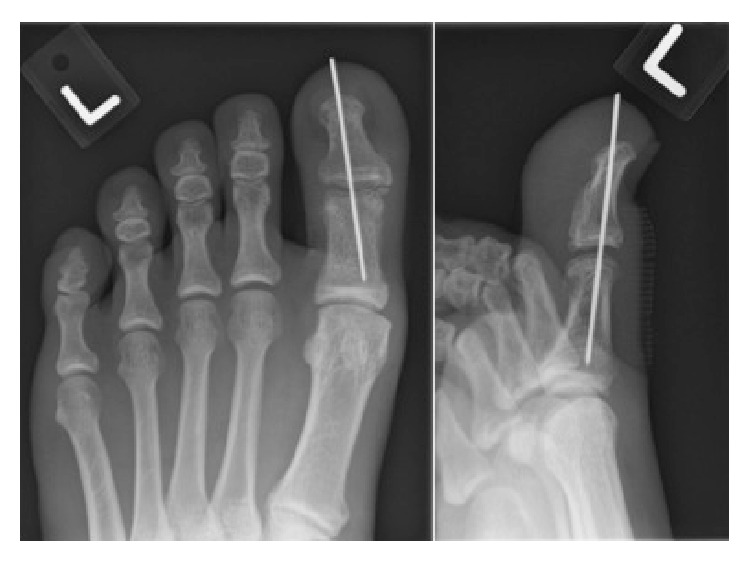
These are the radiographs obtained 2 weeks postoperatively showing an enlocated IP joint of the left great toe with the Kirschner-wire well placed.

The patient was followed up at the Specialist Outpatient Clinic at 2 weeks, 4 weeks, and 6 weeks postoperatively with serial radiographs to ensure enlocation of the left great toe IP joint and to monitor surgical complications. The patient remained well and radiographs showed an enlocated IP joint (Figure 5). The Kirschner-wire was removed in the clinic at 6 weeks postoperatively. The patient was then prescribed with occupational therapy to improve the expected residual stiffness. Subsequently, the patient was seen at 3 months and 6 months postoperatively. He had returned to sports and was symptom-free at the last consult.

## 3. Discussion

Dorsal dislocation of the IP joint of the great toe is a rare injury. The anatomy of the joint makes closed reduction difficult to achieve [15]. Suwannahoy et al. [16] conducted a cadaveric study on 100 fresh great toes to document the appearance, number, size, and location of an intra-articular ossicle found in the IP joint space of the great toe. Radiographic studies of the joint revealed 86% of bony mass representing either the sesamoid bone or an intra-articular ossicle. They found that the bony mass was found on the dorsal surface of the plantar capsule of the IP joint 88% of the time. The challenge in closed reduction is likely to be a result of the interposed plantar capsule with the sesamoid as can be deduced from the work of Suwannahoy et al. [16].

In our patient, the initial radiographs at the emergency department were not as suggestive as subsequent ones in diagnosing the dislocation of the great toe. In fact, this has been well documented in the study by Miki et al. [3]. They reported that in up to 44% of patients, the IP sesamoid is invisible radiographically, leading to difficulty not only in diagnosing sesamoid incarceration but also in confirming a successful reduction. In our patient, the dislocation was initially missed on radiographs at the emergency department as only dorsoposterior and oblique views of the toes were obtained (Figure 1). In the context of this rare condition, we recommend obtaining both dorsoposterior and true lateral views should the clinical findings be suggestive of a great toe dislocation.

Open reduction is necessary once attempts at closed reduction are unsuccessful. Both dorsal [2, 3, 6] and medial approach [11, 12] has been described in the literature. With the dorsal approach, the extensor tendon may be retracted aside, or split like in our patient, with the latter affording better surgical exposure [6].

Miki et al. [3], in their study, described in detail the anatomy of the great toe and its sesamoid. They discovered that (1) the thick plantar plate is separate from the flexor hallucis longus tendon and can easily be displaced into the joint; (2) the sesamoid is almost completely buried within the plantar plate, allowing the sesamoid-plantar plate complex to move as a single unit; (3) the plantar plate is connected to the proximal and distal phalanges by fibrous tissue, which prevents dislocation; (4) the plantar plate is not displaced into the joint space as long as connection to either the proximal or distal phalanx remains intact; and (5) the plantar plate can only be dislocated into the joint space with both proximal and distal phalangeal connections divided. With the anatomy defined as such, IP joint dislocations can then be divided into two groups. In type-I dislocations, the sesamoid-plantar plate complex slips into the IP joint, resulting in slight elongation of the great toe but no significant deformity. In type-II dislocations, the sesamoid-plantar plate complex slips into the plantar aspect of the joint and emerges dorsally with the sesamoid overriding the head of the proximal phalanx, causing a hyperextension deformity of the distal phalanx. Our patient falls into this latter group. Clinical and radiographic examination can be utilized to differentiate the two groups. In type-I dislocations, the distal phalanx is in a neutral position and displays resistance to dorsiflexion and plantar flexion. Radiographically, the sesamoid is visualized within a widened joint space and the phalanges are coaxial in type-II dislocations; the distal phalanx is hyperextended and displays little resistance to dorsiflexion. Radiographically, the sesamoid is located dorsal to the head of the proximal phalanx and the distal phalanx is hyperextended. In both types of injury, reduction is difficult because of intact collateral ligaments, as was seen in our patient when he presented at our clinic.

After open reduction of the IP joint in our patient, the joint displayed increased laxity. This arises from overstretching of the capsule and collateral ligaments at the time of injury [3, 6]. As recommended by Woon [8], we decided on temporary Kirschner-wire immobilization. There have been other methods of stabilization described in the literature such as bulky dressing [2], buddy splinting [11], and immobilization in a short leg cast [3] for up to 4 weeks.

We decided to immobilize the IP joint for up to 6 weeks before removing the wire and starting mobilization. Leung and Wong [6] reported that, on removal of Kirschner-wire, recurrent dislocation is rare and the long-term prognosis is excellent. We hope that this will be true for our patient as well.

Dislocation of the IP joint of the great toe secondary to an incarcerated sesamoid is a rare condition that requires a high index of suspicion. Lateral radiographs are mandatory for diagnosis. Closed reduction is difficult given the inherent anatomy and may only have a chance of success performed acutely. Open reduction and Kirschner-wire fixation are a good option in patients presenting with the subacute or chronic setting.
